# Effects of Polyhedral Oligomeric Silsesquioxane (POSS) on Thermal and Mechanical Properties of Polysiloxane Foam

**DOI:** 10.3390/ma13204570

**Published:** 2020-10-14

**Authors:** Chunling Zhang, Jinrui Zhang, Tianlu Xu, Haofei Sima, Jiazi Hou

**Affiliations:** School of Materials Science and Engineering, Jilin University, Chang Chun 130022, China; clzhang@jlu.edu.cn (C.Z.); jrzhang18@mails.jlu.edu.cn (J.Z.); xutl16@mails.jlu.edu.cn (T.X.); smhf19@mails.jlu.edu.cn (H.S.)

**Keywords:** polysiloxane foam, polyhedral oligomeric silsesquioxane with double bond, cell density, thermal stability, mechanical properties

## Abstract

The thermal and mechanical properties of polysiloxane foam are greatly improved by the addition of acrylolsobutyl polyhedral oligomeric silsesquioxane (MA0701, hereinafter referred to as MAPOSS), which has double bonds. The morphologies and properties of the polysiloxane composite foam were characterized. The average cell diameter of the composite foams decreased, while the cell density increased with increasing MAPOSS. Meanwhile, MAPOSS can enhance thermal conductivity and thermal stability. Thermal conductivity increased by 25%, and the temperature at the maximum weight loss rate increased from 556 °C to 599 °C. In addition, MAPOSS also promoted heterogeneous nucleation by functioning as a nucleating agent, which can increase cell density to improve the mechanical properties. The compressive strength of the composite foam increased by 170% compared with that of pure foam. In the composite, MAPOSS increased the cross-linking density by acting as a physical cross-linking point and limited the movement of the segments.

## 1. Introduction

Polysiloxane foam is a kind of polymeric foam, which consists of a main chain of –Si–O–Si– and organic side chains. Polysiloxane foam has a wide range of applications in aerospace, military, and biomedical fields. Polysiloxane foam can be composed by the reaction between polymethylhydrogensiloxane (PMHS), vinyl-polydimethylsiloxane (V-PDMS), and hydroxyl-terminated polydimethylsiloxane (OH-PDMS) [1]. Traditional polymeric foams possess many advantages such as lightweight, low thermal conductivity, high specific surface area, and specific strength [2,3,4,5]. Compared with traditional polymeric foam, polysiloxane foam possesses excellent performance in terms of low and high temperature resistance, chemical stability, electrical insulation, and additional biological compatibility [6]. A long distance and weak interaction exist between the substituent on the Si in polysiloxane, where Si–O–Si has good flexibility and can vary within the range of 104°–180°; in this regard, Si–O bonds have high bond energy, which is significantly higher than those of C–O, C–C, and C–Si bonds.

Silsesquioxanes are a class of molecules that can be described by the empirical formula RSiO_3/2_, where R represents hydrogen, alkyl, alkenyl, aryl, arylene, or their derivatives. The structures of silsesquioxanes include random, ladder, cage, and partial cage [7]. The first oligomeric organosilsesquioxanes were isolated along with other volatile compounds by Scott [8] in 1946. Since then, silsequioxane chemistry has been studied for more than half a century. However, in the past few years, scholars have focused on silsequioxane with specific cage structures.

**Figure 1:**
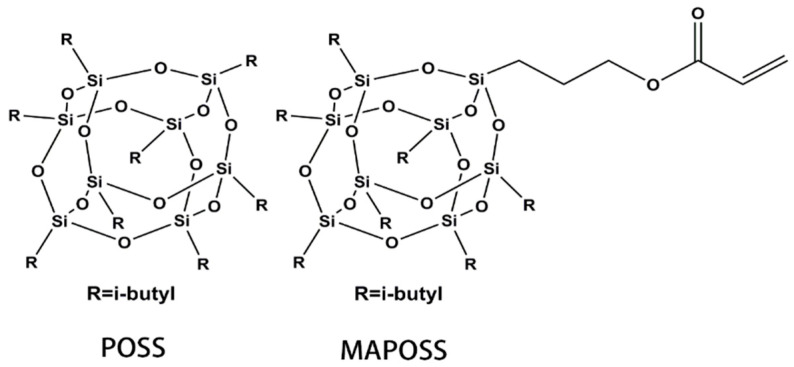
The structure of POSS and MAPOSS.

Polyhedral oligomeric silsesquioxane (POSS), a silsesquioxane compound with a cage structure in the three-dimensional nanoscale has excellent chemical stability and compatibility. POSS is a nanostructured chemical that is approximately 1–3 nm in diameter and can be considered the smallest silica particle. The structure of POSS is shown in Figure 1. POSS compounds embody a hybrid (inorganic–organic) architecture that contains an inorganic framework made up by silicone and oxygen inside, where organic substituents are covered. In contract to silica, each POSS molecule contains organic substituents on its outer surface, which make the POSS nanostructure react with polymers and biological systems. Therefore, reactive or non-reactive groups can be introduced to the structure of POSS such as vinyl-POSS, phenyl-POSS, and amino-POSS. Furthermore, POSS-nanostructured chemicals can be easily incorporated into common plastics via copolymerization, grafting, or blending [9]. Hong et al. [10] reported POSS substituted by phosphorus as a core of constructing new dendritic macromolecules. Hence, POSS has attracted considerable attention due to its application in many areas such as hybrid polymers as well as optical, biomedical, and porous materials [11,12,13,14,15].

In recent years, POSS/polymer nanocomposites have been reported as high-performance organic–inorganic hybrid materials [16,17,18,19,20]. For instance, by comparing the effects of APIB-POSS and AEAPIB-POSS on polyurethane foam, Członka et al. [21] showed that the content of the filling material has a great influence on the foam. Cobos et al. [22] studied the effects of different types and contents of POSS on various properties of PCL-derived composites. Niemczyk et al. [23] studied a new type of POSS and applied it to the flame retardant aspect of PP materials. In this study, the unique structure of POSS with a double bond (MAPOSS) was introduced into the polymer matrix by chemical bonds or van der Waals forces. MAPOSS can be easily composed with the composite by copolymerization, grafting, and so on. According to the structure of polysiloxane foam, MAPOSS was used to prepare polysiloxane composite foams with improved thermal stability and mechanical properties. The double bond contained in MAPOSS makes POSS more conducive to bond formation reactions. In addition, the cage structure of POSS can be used to limit the movement of the polysiloxane segments. On one hand, it makes rearrangement more difficult, which can improve the thermal stability of the foam; on the other hand, it can improve the mechanical properties of the foam. The morphology and the properties of polysiloxane composite foams were systematically characterized, and the related mechanism is discussed in detail.

## 2. Materials and Methods

### 2.1. Materials

Vinyl-polydimethylsiloxane (vi-PDMS), polymethylhydrogensiloxane (PMHS), and hydroxyl-terminated polydimethylsiloxane (OH-PDMS) were supplied by Huazhirun Chemical Industrial Co. Ltd. (Shanghai, China). High performance platinum catalyst was supplied by Nuohai Chemical Plant (Guangzhou, China). MAPOSS was purchased from Hybrid Plastics Co. Ltd. (Hattiesburg, MS, USA).

### 2.2. Preparation of Polysiloxane Form with MAPOSS

**Figure 2:**
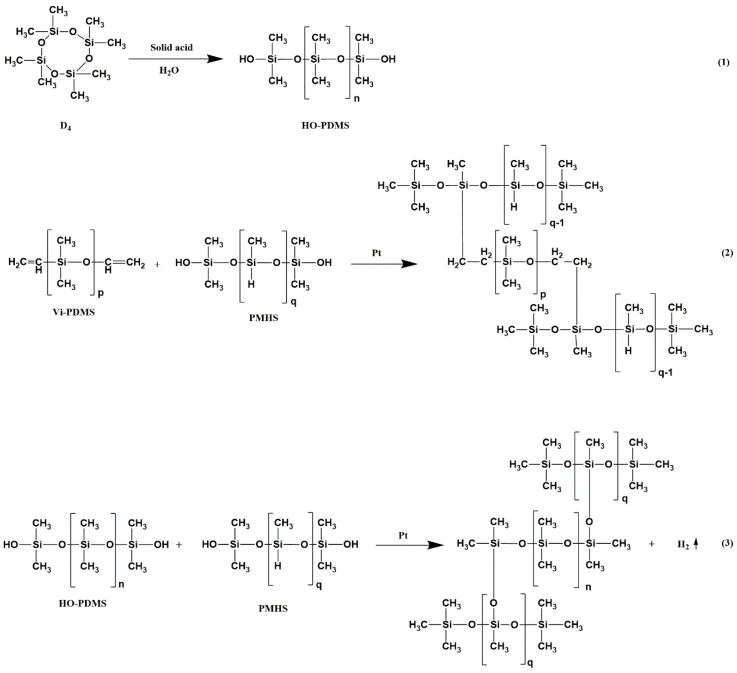
Illustration of the synthesis process of OH-PDMS and polysiloxane foam.

**Figure 3:**
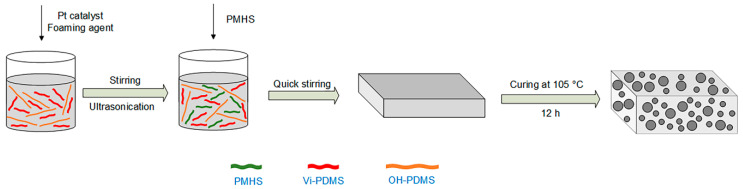
Flowchart for preparation of polysiloxane foam.

Certain amounts of OH-PDMS, vi-PDMS, Pt catalyst, and foaming agent were added to the vessel. After stirring, a certain amount of MAPOSS was added into the composite. The new composite was mechanically stirred for 1 h until a uniform system was formed. Afterward, PMHS was added. PMHS was added last because it can react with other reactants rapidly. This phenomenon had a detrimental influence on the foaming process. After being rapidly mixed, the composite was poured into a mold quickly and curing reaction was performed at a certain temperature. In the end, polysiloxane forms with different MAPOSS content were prepared. The MAPOSS contents were 0, 0.5, 1.0, 1.5, 2.0, and 2.5 wt%, which were marked as S1, S2, S3, S4, S5, and S6, respectively. The reaction equation [24] is shown in Figure 2 and the reaction flow chart is shown in Figure 3.

### 2.3. Characterization

The structural polysiloxane form was characterized by Fourier transform infrared spectroscopy (Bruker TENSOR27, Bruker, Karlsruhe, Baden-Württemberg, Germany) tested in total reflection mode.

The equilibrium swelling degree of the sample was calculated by Equation (1). The sol fraction of the samples was calculated by Equation (2). A series of samples of similar quality was separately immersed in a toluene solution. The samples were collected at intervals to measure the mass whose surface was dried with filter paper. The procedure was repeated several times until the sample quality no longer changed. When the swelling equilibrium was reached, the samples were taken out to dry in the oven after wiping toluene on the surface of the samples. Mass was then measured.

(1) $$Q=\frac{m-m_{0}}{m_{0}}\times 100\%$$

where m_0_ is the mass of the non-swelling sample; and m is the mass of the sample after swelling equilibrium.

(2) $$S=\left(m_{0}-m_{1}\right)/m_{0}\times 100\%$$

where m_0_ is the mass of the non-swelling sample; and m is the mass of the sample after swelling equilibrium.

An environment scanning electron microscope (SEM, FEI XL-30 ESEM FEG, Hillsboro, OR, USA) with an acceleration voltage of 10 kV was utilized to observe the microscopic morphologies of the sample. The cell density of the samples was calculated by Equation (3).

(3) $$N={\left({\mathrm{nM}}^{2}/A\right)}^{\frac{3}{2}}\cdot \frac{\rho_{f}}{\rho }$$

where n is the number of cells in the scanned image; M is the magnification; A is the area of the scanned image; ρ is the density of the polysiloxane matrix; and $\rho_{f}$ is the density of the polysiloxane foam.

A thermal conductivity meter (TPS 2000, Hot-Disk, Uppsala, Sweden) was used to investigate the thermal conductivity of the samples. In the test, the experimental temperature was 20 ± 2 °C and the sample size was 30 mm × 30 mm × 3 mm.

The glass transition temperature of the polysiloxane foam was tested using a differential scanning calorimeter (TA Q20 DSC, New Castle, DE, USA).

A thermogravimetric analyzer (TGA, Pyris 1, PerkinElmer, Waltham, MA, USA) was used under a N_2_ atmosphere and air atmosphere at a heating rate of 10 °C/min to investigate the pyrolysis behaviors of the polysiloxane composites.

The mechanical properties of the sample were measured by a WSM series electronic universal testing machine (WSM-20KN), which was made by Changchun Intelligent Instrument Equipment Co. Ltd. (Chang Chun, China). During the compression test, the sample size was 30 × 15 × 10 mm^3^, and the compression rate was 5 mm/min. Each sample was tested three times, and the results were averaged. The density of the samples was tested according to ISO 845-2006 [25]. The mass of the sample was weighed with an analytical balance, the volume of the sample was measured with a micrometer, and the density of the sample was calculated with $\rho_{f}$ = m/V. Each sample was tested five times and the results were averaged.

## 3. Results and Discussion

**Figure 4:**
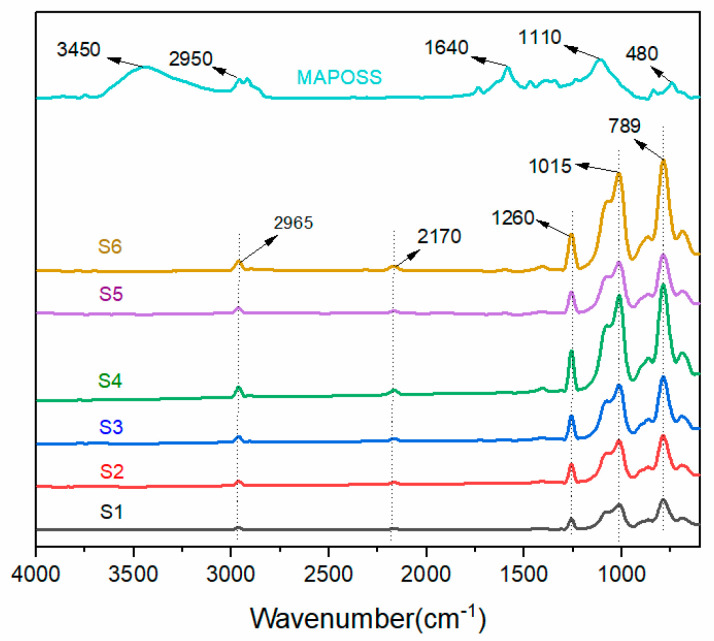
Fourier transform infrared spectrum of polysiloxane foams with different MAPOSS contents.

Figure 4 shows the infrared spectra analysis of polysiloxane foams with different MAPOSS contents. The polysiloxane foam was synthesized successfully with MAPOSS. In Figure 4, the characteristic peak of the double bond in MAPOSS did not appear at 1620 cm^−1^, which indicates that the double bond formed a covalent bond with Si–H. The double bond in the vi-PDMS and the Si–OH in OH–PDMS completely disappeared.

**Figure 5:**
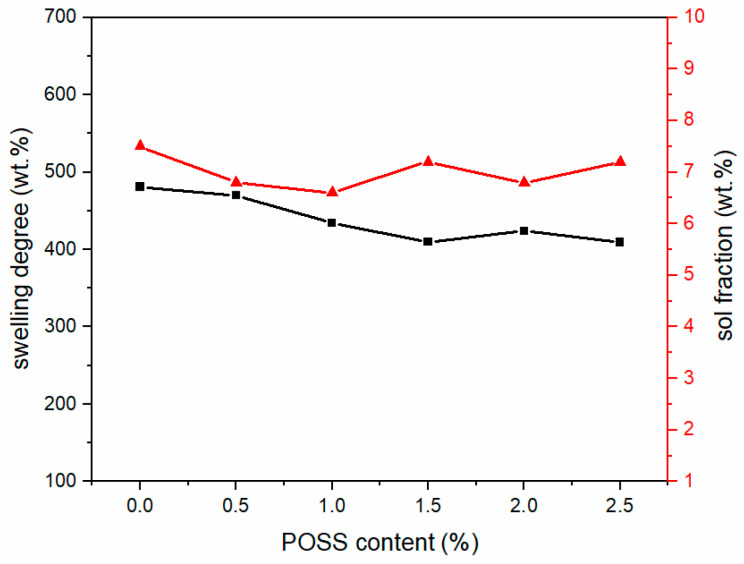
The swelling degree and sol fraction curves of polysiloxane foams with different MAPOSS contents.

Figure 5 shows the equilibrium swelling and the sol fraction curves of the polysiloxane foam. The swelling degree of polysiloxane is inversely proportional to the crosslinking density, which can be seen in Equation (4). When Q increases, $\bar{M_{c}}$ will also increase. $\bar{M_{c}}$ is the average relative mass of the cross-linked polymer between the link points. When the $\bar{M_{c}}$ of polysiloxane decreases, the crosslink density increases.

(4) $$\bar{M_{c}}=\frac{\rho \tilde{v_{1}}Q^{\frac{5}{3}}}{1-\frac{1}{x_{1}}}$$

where ρ is polymer density; $\tilde{v_{1}}$ is molar volume of solvent; Q is the swelling degree; and $x_{1}$ is polymer–solvent interaction parameters.

After the addition of MAPOSS, the cross-link density of the polysiloxane network increased, but the distribution of the network did not change significantly. MAPOSS restricted the movement of the segment to some extent, but had minimal effect on the distribution of the cross-link points. This phenomenon could be due to several factors. First of all, when MAPOSS with the double bond was added to the system, an addition reaction occurred between a part of the double bond and Si–H, which could increase the cross-link density. This phenomenon made MAPOSS easily dispersed in the polysiloxane matrix. In addition, MAPOSS, as a three-dimensional nanoparticle, can be a physical cross-link point, which can also increase the crosslink density. MAPOSS distributed in the middle of the polysiloxane network limited the movement of the siloxane segments, thereby making the segments more entangled. However, MAPOSS cannot change the distribution of the polysiloxane network. The network can be obtained by the addition and polycondensation reaction between linear PDMS. Only a small amount of MAPOSS participated in the reaction, and the distance between adjacent cross-linking points was large. Therefore, MAPOSS has a minimal effect on the distribution of cross-linking points.

**Figure 6:**
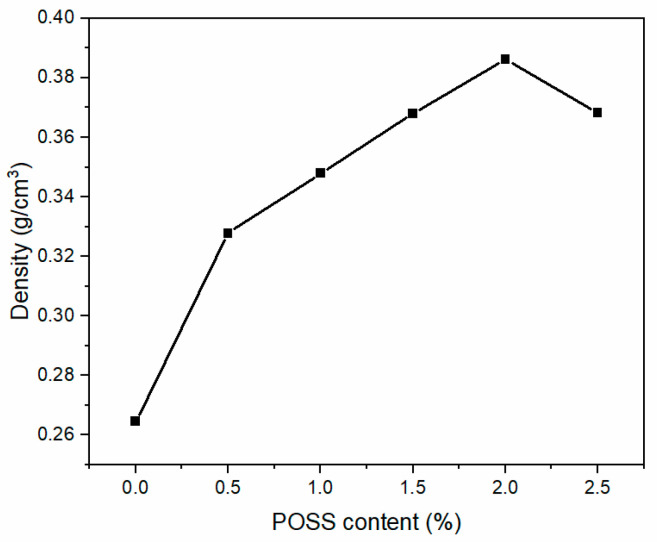
The cell density of polysiloxane foam with different MAPOSS content.

**Figure 7:**
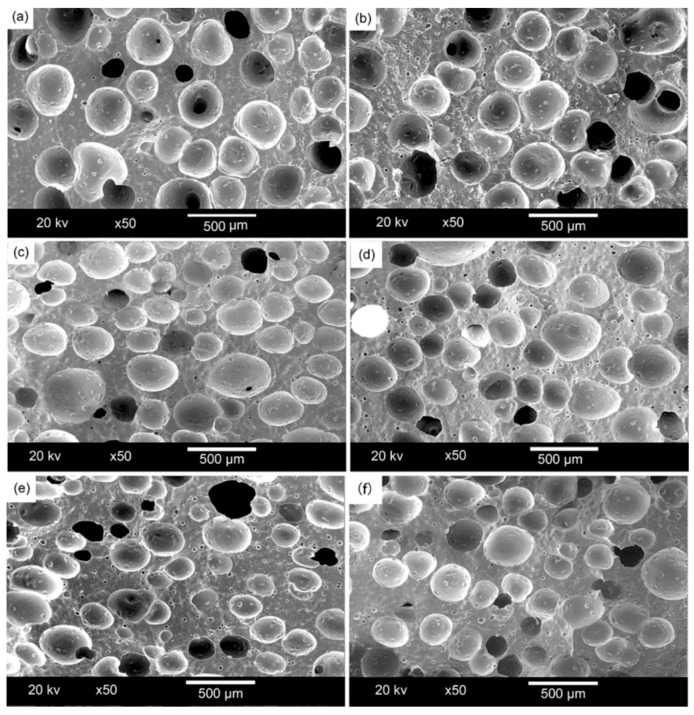
The cell morphology of polysiloxane foam: (**a**) S1, (**b**) S2, (**c**) S3, (**d**) S4, (**e**) S5, and (**f**) S6.

**Figure 8:**
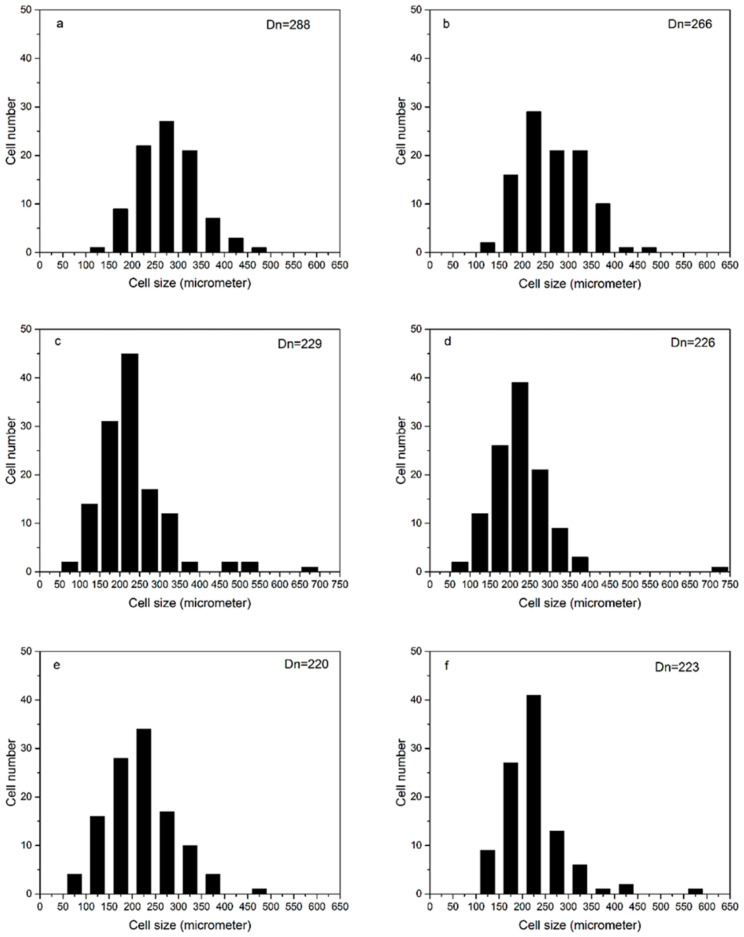
The cell distribution of polysiloxane foam: (**a**) S1, (**b**) S2, (**c**) S3, (**d**) S4, (**e**) S5, and (**f**) S6.

Figure 6 shows the cell density of polysiloxane foams with different MAPOSS contents. As the MAPOSS content increased, the cell density increased from 1.766 × 10^8^ cells·cm^−3^ to 4.937 × 10^8^ cells·cm^−3^. Figure 7 reveals the cell morphology of polysiloxane foams with different MAPOSS contents. As the content of MAPOSS increases, the number of cells increases significantly. Figure 8 shows a diagram of the distribution of polysiloxane foam cells. MAPOSS can change the cell diameter. As the MAPOSS content increased, the average diameter of the foam decreased from 288 μm to 220 μm. The distribution of the cells gradually narrowed and then gradually widened. The upward trend of cell density was most pronounced at 1%. At a low percentage, a part of MAPOSS participated in the bonding reaction, and the other part was uniformly dispersed in the polysiloxane matrix as a nucleating agent, thereby increasing the number of nucleation points that was needed for cell growth. The point reduced the nucleation work of heterogeneous nucleation, which made the nucleation of the cells easy. When excess MAPOSS was added into the matrix, the average diameter of the cells was large, and the cell density declined. In Figure 7, with the increase in MAPOSS content, the cell density increased, and the cell diameter decreased obviously. However, when the content of MAPOSS is too high, the polysiloxane cell morphology begins to deteriorate, and the cell diameter increases significantly. Furthermore, the addition of excess MAPOSS also resulted in poor distribution because excessive MAPOSS easily agglomerated in the polysiloxane matrix, which was not conducive to cell nucleation. Therefore, when the MAPOSS was added to the matrix, it reduced the nucleation work, which makes the matrix easy to nucleate out of phase and improves the cell density.

**Figure 9:**
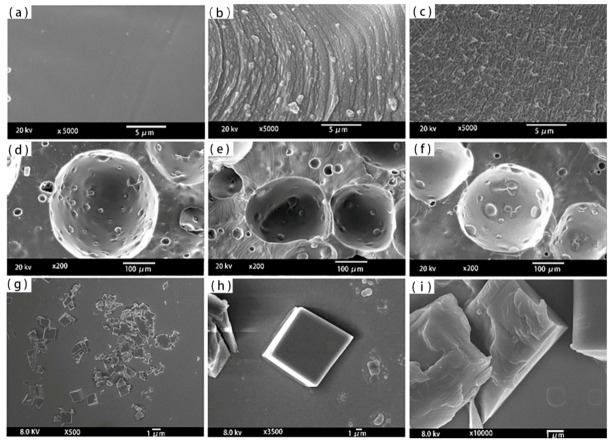
The cell morphology of MAPOSS and polysiloxane foams: (**a**,**d**) S1, (**b**,**e**) S4, (**c**,**f**) S6, and (**g**–**i**) MAPOSS.

Figure 9 shows a photomicrograph of MAPOSS and polysiloxane foam. MAPOSS existed in a block-like crystal state possibly due to the growth of MAPOSS particles and the occurrence of agglomeration. Before adding MAPOSS to the foam, small numbers of white particles were left as residues after decomposition. After adding MAPOSS, many small solid particles that are of non-uniform size can be observed in the gap of the foam. Only part of MAPOSS participated in the bonding reaction, and the rest is uniformly distributed in the matrix. In this process, with the growth of crystal grains, MAPOSS easily agglomerated, leading to a non-uniform particle size. When the MAPOSS content was extremely high, continuous MAPOSS grains were distributed in the gaps. This result may be due to the high MAPOSS content, which could be beneficial to the growth of crystal grains. Thus, the cell morphology was relatively regular. After using MAPOSS, the small holes in the gap increased. leading to incomplete bubble growth. MAPOSS can increase the heterogeneous nucleation efficiency as a nucleating agent in the polysiloxane matrix. This finding is consistent with the above conclusions regarding the increase in cell density.

**Figure 10:**
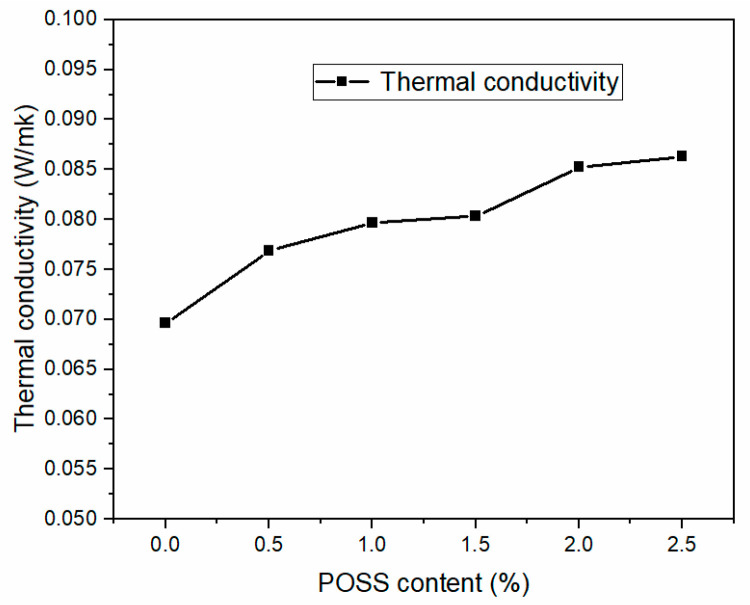
Thermal conductivity of SIFs with different MAPOSS content.

MAPOSS improved thermal conductivity. As is shown in Figure 10, as the MAPOSS content increased, the thermal conductivity of the polysiloxane increased. The thermal conductivity of the foam without MAPOSS was 0.0696 W·mK^−1^, and the maximum thermal conductivity was approximately 0.086 W·mK^−1^ at 2.5 wt%, which improved by 23.5%. Heat transfer was quite complex in the foam materials. Multiple factors influence thermal conductivity such as the density of polysiloxane foam and its microscopic morphology. According to the morphology analysis, as the MAPOSS content increased, the cell density and the average diameter did not show significant change. The addition of MAPOSS did not significantly increase the proportion of gas heat conduction. Conversely, the addition of MAPOSS increased the heat transfer of the solid phase rather than the gas phase. MAPOSS grew into large crystal particles uniformly distributed in the polysiloxane matrix. Therefore, phonon heat conduction may exist [26]. At the interface between MAPOSS and polysiloxane, phonon scattering may occur. This phenomenon is similar to establishing a physical barrier between the delaying phonon heat conduction; the phenomenon is called interfacial thermal resistance, which is used to characterize interfacial heat conduction.

**Figure 11:**
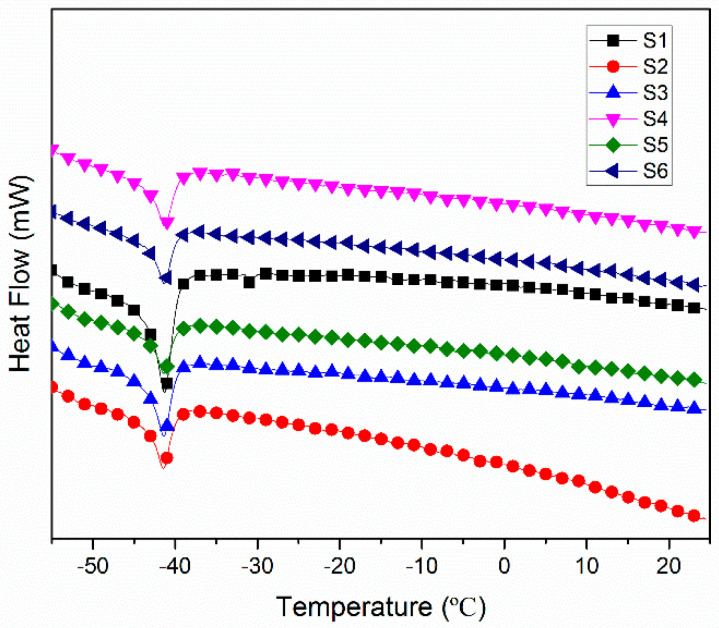
Differential scanning calorimetric curves of polysiloxane foams with different POSS content.

Figure 11 shows the DSC curve for the polysiloxane foam of different MAPOSS contents. MAPOSS cannot change the glass transition temperature of polysiloxane foam. MAPOSS was maintained at a low temperature, indicating that the compound can be used at low temperatures. The glass transition temperature, which determines the temperature domain of the use of polysiloxane-related materials, is an important parameter for the thermal properties of polysiloxane foams. Low temperature resistance is one of the important properties of polysiloxane materials. The glass transition temperature of the polysiloxane foam (approximately −41 °C) did not show substantial change with increasing MAPOSS content. In general, the main factors that affected the glass transition temperature include the degree of freedom of the movement of the polymer network segment, cross-linking, and intertwining among molecular chains. MAPOSS faced difficulty in restricting the movement of polysiloxane chains. On one hand, although MAPOSS is primarily used as a physical cross-link point, which can limit the movement of polysiloxane segments, the main chain still had high flexibility. On the other hand, most MAPOSS were dispersed in the compound, which did not react. This has an extremely limited restriction on the movement of the segments due to the excellent flexibility of the polysiloxane backbone.

**Figure 12:**
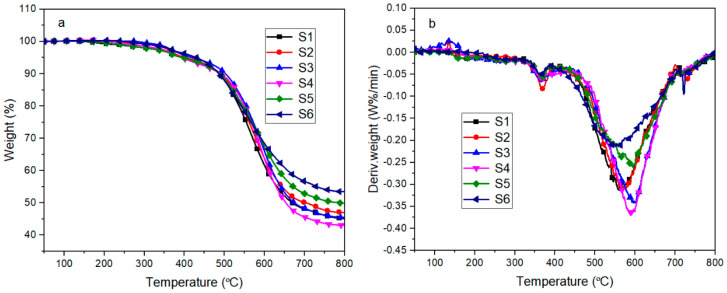
TGA (**a**) and DTG (**b**) curves of SIFs with different MAPOSS content under nitrogen atmosphere.

Figure 12 shows the thermal decomposition curves of polysiloxane foams with different MAPOSS contents in nitrogen. The thermal decomposition curves exhibited three decomposition stages under the N_2_ atmosphere. The first stage occurred at 150 °C to 300 °C due to the decomposition of the residue remaining after the decomposition of the blowing agent and the decomposition of the cyclic by-products produced during the synthesis of linear polydimethylsiloxane. As shown in Figure 12b, the weight loss was minimal mainly because of the addition of a blowing agent at a low content. The second decomposition stage occurred at 300 °C to 450 °C, which was mainly due to the decomposition of the polysiloxane network. MAPOSS was introduced into the polysiloxane system, some of which participated in the chemical reaction, thereby affecting the rearrangement reaction of the polysiloxane network. Moreover, MAPOSS has a three-dimensional cage structure [27], which limited the movement of the polysiloxane segments, resulting in difficulty in the rearrangement reaction [28]. Thus, MAPOSS can improve its initial thermal stability. As shown in Table 1, as the MAPOSS content increased, the initial decomposition temperature increased from 401 °C to 420 °C. However, as the MAPOSS content continued to increase, the initial decomposition temperature began to decrease, indicating that the addition of a small amount of MAPOSS increased the initial thermal stability of the foam. The temperature in the third decomposition stage ranged from 460 °C to 700 °C, which was the main decomposition stage of the polysiloxane network including intermolecular rearrangement and intramolecular rearrangement. The decomposition products may be higher order rings. Another possible mechanism of decomposition is the mechanism of random chain scission. High temperatures favored the movement of molecular segments, which promoted the decomposition of the polysiloxane segments. As shown in Table 1, as the MAPOSS content increased, the temperature at the maximum weight loss rate increased from 556 °C to 599 °C, and the addition of MAPOSS improved the high temperature thermal stability of the silicone foam. MAPOSS also participated in the formation of the cross-linked network, thereby promoting the formation of a thick char layer and providing a barrier [29]. In summary, in addition to a part of the MAPOSS that was involved in the reaction, some of the MAPOSS was distributed in the polysiloxane matrix. These MAPOSS can act as physical cross-linking points, thereby restricting the movement of the polysiloxane segments, hindering their intermolecular and intramolecular action. Thus, the rearrangement reaction led to high temperature thermal stability.

**Figure 13:**
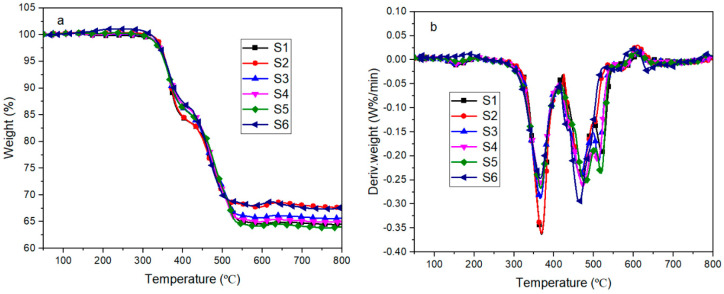
TGA (**a**) and DTG (**b**) curves of SIFs with different MAPOSS content under an air atmosphere.

Figure 13 shows the thermal decomposition curve of silicone foam in an air atmosphere. The silicone foam also exhibited three stages of decomposition. The first and second stages occurred at 150 °C to 230 °C and at 260 °C to 420 °C, respectively. The first stage was mainly the decomposition of the foaming agent and cyclic by-products. A small amount of the blowing agent was present; thus, its quality loss was small. The second stage was mainly the oxidative decomposition of the side chains in the polysiloxane network, which was accompanied by the formation of a large number of long-chain radicals and released H_2_O and CO_2_. The third stage occurred at 420 °C to 550 °C, in which free radicals were generated by the oxidation of the previous stage cross-linked with each other, accompanied by the release of the siloxane fragments. These fragments eventually formed a black product that was difficult to distinguish. According to Table 2, as the MAPOSS content increased, the initial decomposition temperature of the silicone foam did not substantially change. Although MAPOSS has a cage structure, the top corner was found to be a methyl group that can be oxidized easily under the action of air. MAPOSS also cannot produce long-chain free radicals. Thus, the cross-linking reaction between the free radicals in the third stage was not considerably affected. At the same time, the DTG curve showed that MAPOSS did not change the decomposition temperature range.

**Figure 14:**
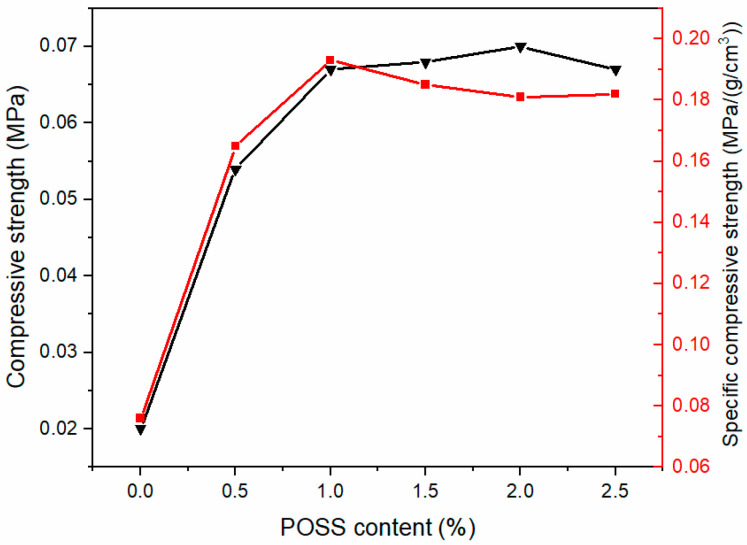
Mechanical strength of SIFs with different MAPOSS content.

Figure 14 shows the mechanical properties of polysiloxane foams with different MAPOSS contents. The compressive strength and specific compressive strength of the polysiloxane foam to which MAPOSS was not added were the smallest (0.02 MPa and 0.076 MPa·(g·cm^−3^)^−1^, respectively). The addition of a small amount of MAPOSS (0.5 wt%) greatly improved the mechanical properties of the silicone foam, and its compressive strength increased by approximately 170%. When the addition amount of MAPOSS reached 2 wt%, the mechanical properties of the polysiloxane foam increased to the maximum, which was 0.07 MPa. While the specific compressive strength reached its maximum at 1 wt%. Therefore, when 1 wt% MAPOSS was added, it had the highest mechanical performance. Many factors affected the mechanical properties of foam materials. On one hand, the density of the foam had a considerable influence on the mechanical properties. On the other hand, the microscopic cell structure of the silicone foam also influenced the mechanical properties. Based on the morphology analysis, with increasing MAPOSS content, the average diameter of foam bubbles gradually decreased, and the density of bubbles gradually increased, thereby increasing the compression strength. MAPOSS itself had a three-dimensional spatial structure, which may harden the solidified polysiloxane matrix. The morphology also showed that MAPOSS was uniformly distributed in the polysiloxane matrix and had a relatively regular grain structure. Moreover, the compression resistance of MAPOSS was higher than that of the polysiloxane matrix. Excessive MAPOSS may be attributed to agglomeration. Under the action of external force, stress temperature at the maximum weight loss rate increased from the concentration that occurred at the location of MAPOSS agglomeration, thereby decreasing the mechanical properties. MAPOSS also restricted the movement of polysiloxane segments as a physical cross-linking point, thereby enhancing the mechanical properties of the silicone foam.

## 4. Conclusions

In this experiment, a MAPOSS/polysiloxane composite foam was prepared by adding MAPOSS with a cage structure to a polysiloxane matrix. In the composite, part of MAPOSS participated in the chemical reaction. Another part of MAPOSS was evenly distributed in the polysiloxane matrix, which can be used as a nucleating agent to reduce nucleation and promote heterogeneous nucleation, thereby reducing the average diameter of the cells of the polysiloxane foam. The minimum diameter of the cell was 220 µm, and the maximum density of the cell was 4.937 × 10^8^ cells·cm^−3^. It also limited the movement of the polysiloxane segment as a physical cross-linking point to increase the cross-link density. The introduction of MAPOSS increased the thermal conductivity of the polysiloxane foam by approximately 23.5% and also improved the thermal stability. However, the thermo-oxidative stability did not change significantly. Adding a small amount of MAPOSS (0.5 wt%) to the polysiloxane foam significantly improved the compressive strength and specific compressive strength. On the whole, when 1 wt% MAPOSS was added, it had the highest mechanical performance. With increasing MAPOSS content, the maximum compressive strength reached 0.07 MPa, and the maximum specific compressive strength was 0.185 MPa·(g·cm^−3^)^−1^. In summary, this foam can be used in many fields. For example, it can be used as filler for aircraft wings and propellers in the aviation field, and it can also be used as a thermal insulation material in missiles in the military. In daily life, it can also be used as a filler for house roofs.

## Figures and Tables

**Table 1:** The thermal stability of polysiloxane foam with different MAPOSS contents.

Sample Code	T_5%_ (°C)	T_max_ (°C)	Residual (%)
S1	401	556	44.8
S2	403	578	47.1
S3	420	599	45.5
S4	394	596	42.9
S5	395	592	49.6
S6	430	557	53.1

**Table 2:** The thermo-oxidative stability of polysiloxane foam with different MAPOSS content.

Sample Code	T_5%_ (°C)	T_max_ (°C)	Residual (%)
S1	356	371	64.6
S2	357	372	67.8
S3	355	374	65.7
S4	356	376	65.1
S5	354	374	64.2
S6	357	379	67.7
